# Adenosine A_2B_ Receptors: An Optional Target for the Management of Irritable Bowel Syndrome with Diarrhea?

**DOI:** 10.3390/jcm6110104

**Published:** 2017-11-03

**Authors:** Teita Asano, Mitsuko Takenaga

**Affiliations:** Institute of Medical Science, St. Marianna University School of Medicine, 2-16-1, Sugao, Miyamae-ku, Kawasaki 216-8512, Japan; m2take@marianna-u.ac.jp

**Keywords:** adenosine A_2B_ receptor, irritable bowel syndrome, visceral hypersensitivity, diarrhea, abdominal pain, immune response

## Abstract

Irritable bowel syndrome (IBS) is a functional gastrointestinal disorder, with the characteristic symptoms of chronic abdominal pain and altered bowel habits (diarrhea, constipation, or both). IBS is a highly prevalent condition, which negatively affects quality of life and is a significant burden on global healthcare costs. Although many pharmacological medicines have been proposed to treat IBS, including those targeting receptors, channels, and chemical mediators related to visceral hypersensitivity, successful pharmacotherapy for the disease has not been established. Visceral hypersensitivity plays an important role in IBS pathogenesis. Immune activation is observed in diarrhea-predominant patients with IBS and contributes to the development of visceral hypersensitivity. Adenosine is a chemical mediator that regulates many physiological processes, including inflammation and nociception. Among its receptors, the adenosine A_2B_ receptor regulates intestinal secretion, motor function, and the immune response. We recently demonstrated that the adenosine A_2B_ receptor is involved in visceral hypersensitivity in animal models of IBS. In this review, we discuss the possibility of the adenosine A_2B_ receptor as a novel therapeutic target for IBS.

## 1. Introduction

Irritable bowel syndrome (IBS) is a chronic functional gastrointestinal disorder involving abdominal pain or discomfort, bloating, diarrhea, and/or constipation [1]. IBS is defined by the symptom-based Rome criteria without the detection of organic causes [2,3]. The prevalence of IBS in the general population is remarkably high (affecting approximately 11% of the world’s population) [3]. Although IBS is not life-threatening, it accords a large burden on global healthcare and markedly reduces quality of life [4]. Four subtypes of IBS are recognized, depending on the predominant stool pattern: IBS with constipation (IBS-C), IBS with diarrhea (IBS-D), mixed IBS (IBS-M), and un-subtyped IBS (IBS-U) [2,3]. Pharmacological treatments effective against abdominal pain are insufficient in IBS-D therapy, and drugs targeting chemical mediators or receptors involved in visceral sensitivity have recently been developed. Alosetron and ramosetron, both antagonists of the serotonin 3 (5-hydroxytryptamine 3; 5-HT_3_) receptors, are approved for use in patients with IBS [5,6]. Rifaximin, an antibacterial drug, and eluxadoline, which has both μ-opioid receptor agonist and δ-opioid receptor antagonist activities, were also recently approved for IBS-D treatment [6,7]. However, although these advances have significantly improved IBS-D treatment, additional pharmacological therapeutic options are required [8].

The mechanism of IBS pathogenesis is not fully understood. However, visceral hypersensitivity plays a vital role in its development [9]. Therefore, drugs targeting visceral hypersensitivity could represent a novel pharmacological approach to IBS treatment. Enhanced visceral perception, psychological factors, altered intestinal microbiota, and immune activation all contribute to visceral hypersensitivity [10,11,12]. In particular, immune activation in the gastrointestinal tract has recently attracted much attention as a potential mechanism. This is because inflammatory mediators in the intestinal mucosa, such as adenosine 5′-triphosphate (ATP), bradykinin, and adenosine, activate primary afferent nerve endings. This indirectly triggers the release of nociceptor ligands such as histamine and 5-HT, or causes increased intestinal permeability, which allows exposure to bacteria or their products [12,13]. These harmful events enhance visceral sensitivity. Among inflammatory cells, mast cells appear to be key players. This suggests that anti-inflammatory agents might be a promising therapeutic strategy for visceral hypersensitivity.

Adenosine is a purine nucleoside metabolized from ATP. Among its receptor subtypes, the A_2B_ receptor has low affinity and is activated by high concentrations of adenosine arising from pathological conditions [14]. Until recently, our understanding of this receptor was limited by the lack of pharmacological and molecular tools, including genetically modified mouse models. However, progress in developing these tools has revealed multiple functions, including immunomodulation, relaxation of smooth muscle, and intestinal secretion, in various cell types [14,15]. Recently, we suggested that blockade of the A_2B_ receptor could be therapeutically beneficial for IBS-D, based on the attenuation of visceral hypersensitivity and reduced stress-induced defecation in animal models [16]. The presence of functional A_2B_ receptors has been demonstrated in various tissue types. Furthermore, A_2B_ receptors have been found to be potential therapeutic targets in several diseases. In this review, we focus on the role of the A_2B_ receptor in gut function and the immune response, and its involvement in IBS pathogenesis, particularly in the control of intestinal secretion, motility, and sensation.

## 2. Etiology of IBS

Regardless of the subtype, visceral hypersensitivity is a common feature of patients with IBS. It is thought that increased visceral sensitivity predominantly contributes to the abdominal pain experienced in IBS. In fact, patients with IBS have a lowered threshold for abdominal pain in response to colorectal distension (CRD) compared with healthy control individuals [9]. Stimulation of visceral sensitivity in the gastrointestinal tract might be linked not only to abdominal pain, but also to other symptoms, including diarrhea, as chemical mediators facilitating visceral sensitivity such as 5-HT and ATP can also directly or indirectly activate secretion and sensorimotor functions. IBS is defined as a functional gastrointestinal disorder without any obvious abnormalities. However, increasing evidence indicates that low-grade inflammation is important for IBS symptoms and visceral hypersensitivity. In particular, low-grade inflammation is consistently observed in patients with IBS-D.

## 3. Visceral Hypersensitivity

Visceral hypersensitivity is a multifactorial process. Recent studies have revealed the presence of low-grade mucosal inflammation and immune activation, related with impaired epithelial barrier function (increased paracellular permeability) and aberrant neuronal sensitivity in patients with IBS [11,13,17]. Chemical mediators from inflammatory cells sensitize primary afferent nerves, favoring the recruitment of silent nociceptors, which results in secondary spinal sensitization. The release of mediators such as potassium ions, ATP, bradykinin, and prostaglandin E_2_ (PGE_2_) directly activates nerve endings and indirectly triggers the release of algesic mediators such as histamine, 5-HT, and nerve growth factor from other cells. These mediators in turn stimulate proximal afferent nerve endings and nociceptors, leading to pain sensitization or diarrhea. In particular, consistent observations in human and animal studies support an important role in mast cells and their mediators in exciting visceral sensory neurons [17,18,19].

## 4. Immune Activation in IBS

Evidence of immune activation in patients with IBS has been obtained from blood samples and intestinal biopsies [20,21,22,23,24]. Consistent with this, previous digestive infections and long-lasting inflammatory features are often observed in patients with IBS (termed post-infectious IBS and post-inflammatory IBS, respectively) [25,26]. Although a variety of inflammatory cell types are involved in visceral hypersensitivity, a recent meta-analysis has suggested that increased numbers of mast cells and CD3^+^ T cells in the intestinal mucosa are reliable markers of immune activation in IBS [24]. Mucosal inflammatory cells change the luminal environment to an inflammatory state. Supernatants of colonic biopsies from patients with IBS display increased levels of proteolytic activity, which depends on the activation of nuclear factor (NF)-κB [22]. In addition to local inflammation in the intestinal mucosa, systemic inflammatory changes have been reported in patients with IBS [21,23]. Higher levels of plasma interleukin (IL)-6 and IL-8 have been observed in patients with IBS than in control individuals [21]. In particular, elevated IL-6 levels correlate significantly with IBS symptoms, including abdominal pain and bloating [21,27]. Elevated levels of cytokines and chemokines, including tumor necrosis factor (TNF)-α, IL-1β, and IL-6, were observed in peripheral blood mononuclear cell supernatants from patients with IBS-D, and their levels correlated with the frequency and intensity of abdominal pain [23]. These supernatants caused mechanical hypersensitivity in colonic afferent nerve endings, and this was suppressed by infliximab, a monoclonal antibody against TNF-α. These findings demonstrate that immune activation in patients with IBS is associated with clinical outcomes, and that anti-inflammatory agents can be effective in the treatment of IBS. However, a recent clinical study showed that the anti-inflammatory agent 5-aminosalicylic acid (mesalazine) did not improve abdominal pain or stool consistency in patients with IBS-D compared with placebo groups, nor did it provide satisfactory relief [28].

Increasing evidence suggests that mast cells located at the host-environment interface, in close proximity to sensory nerves, play a significant role in abdominal pain or discomfort. Mast cell mediators such as histamine, tryptase, leukotriene, 5-HT, and platelet-activating factor can activate sensory nerves [17,29]. Sustained sensitization by such mediators could lead to the enhancement of visceral sensitivity. It has been demonstrated that a high density of degranulated mast cells exists in the colonic mucosa of patients with IBS, and the proximity of activated mast cells to mucosal nerve fibers is correlated with the severity of abdominal pain [29]. These observations have suggested that mast cell–nerve interactions are a therapeutic target for visceral pain. Clinical trials have recently been conducted on drugs targeting mast cells. Inhibition of the histamine H_1_ receptor by ebastine reduces visceral hypersensitivity, global symptoms, and abdominal pain in patients with IBS [30]. Furthermore, the mast cell stabilizer ketotifen increases the threshold for discomfort during IBS with visceral hypersensitivity and improves IBS symptoms, including abdominal pain [31]. However, no relationship has been observed among mast cell number, visceral perception, and IBS symptoms.

Although further studies are required to confirm that immune activation is associated with alterations in gut function—particularly visceral sensitivity and IBS symptoms—taken together, the available literature provides valuable insights into the potential mechanisms underlying IBS.

## 5. The Adenosine A_2B_ Receptor

Adenosine is an extracellular purine nucleoside signaling molecule responsible for diverse actions in the nervous, cardiopulmonary, renal, and gastrointestinal systems [32]. It is produced by the dephosphorylation of its precursor, ATP. Under stressful or injurious conditions, ATP is released from cells and is rapidly degraded to adenosine by sequential hydrolysis. Extracellular ATP and adenosine diphosphate (ADP) are mainly hydrolyzed by ecto-apyrase to adenosine monophosphate (AMP), which is further metabolized by ecto-5′-nucleotidase (CD73). The biological actions of adenosine are mediated by binding to G protein-coupled receptors, classified into four subtypes: A_1_, A_2A_, A_2B_, and A_3_. The regulation of biological functions by adenosine is strictly related to its extracellular concentration. A_1_, A_2A_, and A_3_ receptors possess high-affinity ligand-binding characteristics and are activated by physiological adenosine concentrations in the submicromolar range (10–200 nM), whereas A_2B_ receptors are activated by micromolar levels of adenosine (10–100 μM), which are achieved in pathological conditions, including hypoxia, ischemia, and inflammation, or in other stressful environments [32].

Adenosine A_2B_ receptors are expressed in a wide variety of cell types and exert tissue- and cell-specific effects [32,33]. A_2B_ receptor activation triggers the stimulation of adenylate cyclase and phospholipase C by coupling to Gs and Gq proteins, respectively. This stimulation leads to increases in intracellular cyclic AMP (cAMP) and calcium ion levels [32,33]. Transcriptional induction of the A_2B_ receptor is caused by hypoxia, bacterial lipopolysaccharide, or inflammatory mediators, including PGE_2_, IL-1β, and TNF-α [32]. The presence of functional A_2B_ receptors has been demonstrated in hematopoietic, mast, myocardial, intestinal epithelial, muscle, retinal pigment epithelial, endothelium, and neurosecretory cells [15,32,33]. The functions of A_2B_ receptors have highlighted their promise as therapeutic targets for asthma, inflammatory bowel diseases, respiratory diseases, and arteriosclerosis [14,32,34]. The recent development of pharmacological and molecular tools, including genetically modified mice, has advanced our understanding of the roles of the adenosine A_2B_ receptor in pathophysiological processes, including intestinal inflammation [32,34,35]. Previous studies have demonstrated significant A_2B_ receptor involvement in the control of intestinal secretion, motility, and sensation [14,33]. In the following section, we focus on the roles of the A_2B_ receptor in gut function and immune responses associated with IBS pathogenesis.

## 6. Intestinal Fluid Secretion

In the alimentary tract, adenosine A_2B_ receptors are predominantly expressed in the colon, particularly in intestinal epithelial cells and enteric neurons [14]. In the intestinal epithelia, adenosine mediates chloride secretion via the A_2B_ adenosine receptor, which results in the movement of isotonic fluid into the lumen [36]. This physiological process naturally serves to hydrate the mucosal surface and contributes to mucosal defenses against luminal pathogens. However, the extreme secretory flush also produces secretory diarrhea. Studies performed using human intestinal epithelial cells suggest that the stimulation of chloride secretion through apical A_2B_ receptors is a potential target for the treatment of diarrheal diseases [36]. The activation of the A_2B_ receptor by adenosine or agonists produces electrogenic chloride secretion in a polarized human intestinal epithelial cell line [36]. In addition, adenosine A_2B_ receptor-deficient mice display reduced stool water content, suggesting a role of the receptors in chloride secretion [37]. CD73 knockout mice exhibit a defect in converting ATP into adenosine, suggesting that the stimulation of colonic water movement by luminal ATP involves metabolized adenosine and apical A_2B_ receptors [38]. Furthermore, bacterial toxins trigger the chloride secretion response through the A_2B_ receptor. The A_2B_ receptor antagonist MRS-1754 prevents the stimulation of secretion by conditioned medium containing ATP released from cells infected with enteropathogenic *Escherichia coli* [39]. Clostridium difficile toxins upregulate the expression of the A_2B_ receptor in the human colon cell line HCT-8, and the receptor antagonist ATL-692 decreases toxin-induced secretion [40]. These observations indicate that the adenosine A_2B_ receptor regulates intestinal fluid secretion in epithelial cells, and that excess activation could cause diarrhea.

## 7. Release of 5-HT from Enterochromaffin Cells

Enterochromaffin (EC) cells are neuroendocrine cells in the epithelial lining of the gastrointestinal tract lumen. These cells secrete 5-HT and modulate gut motility, secretion, and pain. EC cells express the adenosine A_2B_ receptor and are thought to function as mechanosensors. A recent study demonstrated that the adenosine receptor agonist 5′-*N*-ethylcarboxamidoadenosine (NECA) stimulates 5-HT release, while the antagonist MRS-1754 inhibits its secretion from EC cells [41]. Moreover, stimulation using a flex-based mechanical stress model induced the release of 5-HT, and this was inhibited by MRS-1754 and amplified by NECA [41]. The release of 5-HT induced by mechanical stretch might be involved in A_2B_ receptor function. This is supported by the finding that A_2_ receptor antagonists suppressed mechanically evoked 5-HT release in the human carcinoid cell line, BON cells, suggesting that endogenous adenosine can activate excitatory A_2A_/A_2B_ receptor signaling pathways in EC cells to regulate 5-HT release [42,43]. Another study showed that the A_2B_ receptor localizes in human epithelial cells lining both the villi and crypts of the intestine [44]. The A_2B_ receptor has also been found to be overexpressed in EC cells from patients with diseases such as Crohn’s disease [41,43]. Thus, the A_2B_ receptor might be expressed in human EC cells and induced by inflammatory stress, which modulates 5-HT release.

## 8. Colonic Motility

In addition to regulatory roles in intestinal epithelial cells, the A_2B_ adenosine receptor participates in the modulation of colonic motor function. Nonadrenergic noncholinergic (NANC) inhibitory neurotransmission plays a pivotal role in colonic motility. ATP and nitric oxide (NO) are major mediators of NANC transmission. Recent experiments using A_2B_ receptor-knockout/β-gal-knock-in mice and ATL-801, an A_2B_ receptor antagonist, demonstrated that A_2B_ receptors expressed on enteric neurons regulate distal colonic motility [37]. That study revealed a constipation-like phenotype, with delayed colonic transit, impaired colonic motility, and increased stool retention [37]. Mechanistically, the phenotype might be caused by defects in colonic relaxation, through aberrant A_2B_ receptor activation via the NO-cyclic guanosine monophosphate (cGMP) pathway. Subsequently, another group demonstrated that A_2B_ receptors are located in the neuromuscular compartment, and that the A_2B_ receptor antagonist MRS-1754 enhances both electrically and carbachol-induced cholinergic contractions in normal longitudinal muscles [45]. This result suggests that the activation of A_2B_ receptors is associated with the inhibition of excitatory cholinergic pathways. Although this differs from the findings of Chandrasekharan et al., suggesting that the A_2B_ receptor contributes to the facilitation of inhibitory nitrergic nerve control [37], both observations result in similar inhibitory modulation in the colon. Furthermore, we recently found that MRS-1754 suppresses defecation induced by wrap-restraint stress in normal rats and by novel environmental stress in maternally separated rats [16]. These findings indicate that the specific inhibition of A_2B_ receptors could be used as a therapeutic approach for diarrhea or motility disorders, but further investigations are needed to fully understand the function of the receptor.

## 9. Modulation of the Inflammatory Response

The extracellular accumulation of adenosine contributes to the regulation of inflammation. A_2B_ receptors regulate the immune response in various cell types, including mast cells, epithelial cells, and macrophages. Here, we describe the roles of the A_2B_ receptor in the inflammatory response related to IBS pathogenesis.

Intestinal adenosine A_2B_ receptors modulate inflammation in both acute and chronic models of colitis [46,47,48,49]. Genetic ablation or pharmacological blockade of the A_2B_ receptor with ATL-801 attenuated the clinical and histological features of intestinal inflammation in experimental colitis, in parallel with reductions in IL-6 and keratinocyte-derived chemokine secretion [46,47]. In contrast, the A_2B_ antagonist PSB-1115 promoted intestinal inflammation in an acute colitis model, and this was related to the modulation of IL-10 production [49]. The enhancement of the inflammatory response observed with the loss of the A_2B_ receptor might depend on the protective effects of intestinal epithelial cells rather than immune cells. Recently, it was reported that intestinal epithelial-specific A_2B_ receptors confer protective roles against intestinal inflammation [48]. The reason for the disparate results of these studies remains unclear.

Mast cells are increasingly appreciated as a crucial cell type in initiating inflammation and the immune response. As described above, activated mast cells in the intestinal mucosa play an important role in the pathogenesis of IBS. Adenosine is known to potentiate the antigen-induced degranulation of mast cells, based on the observation that inhaled adenosine causes bronchoconstriction in patients with asthma [50]. There is evidence that enprofylline and theophylline, two clinically used anti-asthmatic agents, block adenosine-induced IL-8 production by the human mast cell line HMC-1 by targeting the A_2B_ receptor, attracting increased interest in this receptor [51]. Moreover, A_2B_ receptors stimulate the release of IL-4, IL-13, vascular endothelial growth factor, and IL-8 from mast cells [52,53]. Conversely, the genetic deletion of the A_2B_ receptor in mice enhances antigen-induced mast cell degranulation [54].

Although we have described the anti-inflammatory actions of the A_2B_ receptor, it is difficult to reconcile the evidence into either pro-inflammatory or anti-inflammatory roles for the receptor, as A_2B_ receptor knockout mice appear to have a pro-inflammatory phenotype compared to wild-type controls, characterized by elevated plasma TNF-α levels and increased vascular permeability for albumin in the colon, kidneys, and lungs [55]. This discrepancy in receptor function may be derived from differences in tissue type (lung or intestine), inflammation type (pro-inflammatory or allergic), or the time scale over which inflammatory response progression was observed (acute or chronic).

## 10. Anti-Nociceptive Actions

Studies on pain sensitivity in relation to the A_2B_ receptor have been limited due to a lack of selective pharmacological tools. However, several studies have recently demonstrated that pharmacological blockade of the A_2B_ receptor is effective in inflammatory pain models. In a formalin-induced pain model, systemic or local administration of the selective A_2B_ receptor antagonist PSB-1115 attenuated nociception in both the first and second phases of the test [56]. Moreover, this inhibitor produced robust dose-dependent analgesia and enhanced morphine-induced anti-nociception in a hot plate test [57]. In addition to effects on the somatic pain response, our study showed that A_2B_ receptor antagonists affect visceral hypersensitivity in response to CRD. We found that the systemic administration of MRS-1754 and PSB-1115 suppressed this pain response in rats [16]. In addition to acute pain, adenosine and the adenosine A_2B_ receptor play major roles in chronic pain by promoting immune–neuronal interaction. A recent study using adenosine deaminase knockout or complete Freund’s adjuvant-injected mice revealed that sustained elevated extracellular adenosine activates adenosine A_2B_ receptors on myeloid cells, which in turn transactivates the nociceptors of sensory neurons, causing neuronal hypersensitivity and chronic pain [58]. These findings imply that the inhibition of the adenosine A_2B_ receptor can suppress pain responses.

## 11. Conclusions and Remarks

Visceral hypersensitivity plays a crucial role in IBS pathogenesis. Previous studies have demonstrated altered inflammatory responses in IBS, and immune activation is likely to be responsible for the stimulation of visceral sensitivity and clinical outcomes. Consistent observations suggest that mast cells are key players in the development of visceral hypersensitivity. Conversely, the adenosine A_2B_ receptor regulates both intestinal fluid secretion into the lumen and colonic motility. These actions can be beneficial for diarrhea in IBS. Although the A_2B_ receptor is involved in the immune response, it is not clear whether it plays a pro-inflammatory or anti-inflammatory role in the gastrointestinal tract. It is also controversial whether the A_2B_ receptor can stimulate mast cell activity. Importantly, blocking the A_2B_ receptor suppresses both somatic and visceral pain. Taken together, although the inhibition of the A_2B_ receptor might be effective for the treatment of pain and diarrhea associated with IBS, with the exception of our recent work, the role of the A_2B_ adenosine receptor in IBS development has not been investigated. Further research is warranted to evaluate the possibility of targeting the A_2B_ receptor for IBS-D treatment.
